# Methylomic Analysis of Nasal Brushings Reveals Two Subgroups in Pediatric Acute Respiratory Distress Syndrome

**DOI:** 10.21203/rs.3.rs-8042030/v1

**Published:** 2026-01-22

**Authors:** James G. Williams, Akhilesh Kaushal, Nirmeen Elmadany, Rashika Joshi, Rhonda Jones, Nathan Gregor, Patrick Lahni, Steven W. Standage, Brian M. Varisco

**Affiliations:** 1 Division of Critical Care Medicine and Department of Pediatrics, University of Arkansas for Medical Sciences, Little Rock, AR, USA.; 2 Arkansas Children’s Hospital, Little Rock, AR, USA.; 3 Department of Pediatrics University of Cincinnati, College of Medicine and Division of Critical Care Medicine, Cincinnati Children’s Hospital Medical Center, Cincinnati, OH, USA.

## Abstract

Pediatric acute respiratory distress syndrome (PARDS) is a significant cause of mortality in the pediatric intensive care unit (PICU), and supportive care remains the mainstay of treatment. The biological heterogeneity of PARDS hampers the development of new therapies. One source of heterogeneity is the epigenetic regulation of gene expression via methylation. We hypothesized that PARDS patients could be classified into at least two subgroups defined by differential methylation of immune-related genes. We conducted a prospective, single-center cohort study of PARDS and control patients under 18 years of age admitted to the PICU. Nasal brushings were obtained on day 1 for methylomic analysis, and clinical information and outcomes were recorded until discharge. We identified two groups of PARDS subjects using PCA and hierarchical clustering, which were defined by the differential methylation of promoters and bodies of genes involved in immune, repair, and regeneration processes. One group trended toward worse clinical outcomes. The other group had a methylation pattern very similar to control subjects. PARDS patients can be divided into two subgroups based on patterns of differential methylation around genes involved in immune, repair, and regeneration processes. These findings, if confirmed, could represent potential targets for future therapies.

## Introduction:

Pediatric acute respiratory distress syndrome (PARDS) is a common indication for admission to the pediatric intensive care unit and is defined by hypoxemia and lung infiltrates on chest x-ray within 7 days of an acute infection, injury, or other insult.^1^ Despite the significant improvement in the overall mortality rate among patients admitted to the PICU over the last several decades,^2^ the mortality rate for PARDS remains high at up to 30% in the most severe cases.^3^ This high mortality rate is in part due to the lack of therapies that improve outcomes for PARDS patients. Despite decades of research, current therapies remain supportive, including the use of lung-protective ventilation^4^ and treatment of any underlying infections. One of the most significant contributors to the many failed interventional trials in PARDS is the vast differences in pathobiology encompassed by syndromic definitions of disease. The differing pathobiology’s within PARDS are caused by the complex interplay of the many varied insults that lead to PARDS and the heterogeneity in the host response to lung injury. To develop new therapies that improve outcomes for PARDS patients, the biological basis for the observed heterogeneity must be discovered.

Efforts to improve our understanding of the heterogeneity have primarily focused on using plasma biomarkers to define subgroups within ARDS.^5–8^ These studies have advanced our knowledge of the subgroups within ARDS but have not elucidated the biology responsible for these subgroups. This is likely because plasma biomarkers can originate from organs affected by systemic processes that are often comorbid with ARDS^9,10^ and do not always accurately reflect the biological processes occurring in the lung.^11^ To better understand the lung-specific biology that accounts for the existence of subgroups within ARDS, we could use lung-specific samples. In most cases, this requires a bronchoscopy, an invasive procedure that involves obtaining a fluid sample from the lungs. This is a significant limitation because bronchoscopy is an invasive procedure and is not always clinically indicated in ARDS patients, particularly in pediatric patients.

Sampling of the nasal epithelium has been a successful approach as it reflects lung-specific biology while avoiding the invasive techniques required to sample the lung directly. This approach has been used successfully in other heterogeneous pulmonary diseases, including pediatric asthma,^12,13^ COPD,^14,^ and lung cancer.^15^ We have previously described the multi-omic analysis of nasal brushings in PARDS subjects and found that there are two subgroups of PARDS patients that differ in their methylation pattern around genes regulating inflammation and the innate immune response.^16^ The group characterized by hypomethylation of promoters adjacent to genes governing the immune response included all patients in our cohort who were immunocompromised and all subjects who died, although this did not reach statistical significance. This work suggested that methylation patterns could distinguish between PARDS subjects with a high risk of poor outcomes and those with a low risk, and that this difference would be due to the methylation patterns of promoters and cis-elements involved in regulating the immune response. Our previous study used a time and data-intensive enzymatic methyl-seq approach that would be difficult to translate. Thus, the goal of this study was to confirm our earlier findings on a new microarray platform with more translational potential using a combination of new patient samples and residual samples from our previous work.

## Methods:

### Human Subjects Research and Ethical Considerations:

This study was conducted at Cincinnati Children’s Hospital Medical Center (CCHMC) Pediatric Intensive Care Unit (PICU), a quaternary pediatric intensive care unit. The study was approved by the CCHMC Institutional Review Board (IRB) on November 28, 2017 (IRB 2015–8514 and 2017–1345). The trial was registered with ClinicalTrial.gov (NCT#03539783). All procedures and protocols were conducted in accordance with the ethical standards established by the CCHMC IRB and the Helsinki Declaration.

### Subjects and Enrollment:

The enrollment period for this study ran from April 2, 2018, to June 30, 2022. The census of the CCHMC PICU was screened daily for eligible patients. We enrolled patients into two groups: PARDS and control. For PARDS subjects, the inclusion criteria were admission to the CCHMC PICU, age less than 18 years, mechanical ventilation, and a diagnosis of PARDS according to the 2015 PALICC criteria.^17^ Exclusion criteria for PARDS subjects were a baseline oxygen requirement greater than 2L per minute and an order of limited resuscitation. The inclusion criteria for control subjects were admission to the CCHMC PICU with an expected length of stay of more than 7 days and no apparent lung disease on admission. The exclusion criteria were the same as those for PARDS subjects. Our control subjects were PICU patients without lung disease expected to be intubated for at least 3 days. The most common operations in our cohort were airway reconstruction, neurosurgical procedures, and total pancreatectomy with auto-islet transplant (TPIAT). Once subjects were identified as eligible, their guardians were approached, and informed consent was obtained before any specimen or data collection took place. Subject assent was obtained as appropriate.

#### Protocol and Specimen Collection:

After consent was obtained, a nasal inferior turbinate brushing was obtained within 24 hours of PARDS diagnosis for PARDS subjects or within 24 hours of admission to the PICU for control subjects. The brush heads were cut off and preserved in RNAProtetect (Qiagen) at −80°C until DNA extraction. A combination of new specimens and older specimens with adequate residual DNA from our previous work were used.

#### Clinical Variables and Outcomes:

Applicable clinical data, including demographic information, comorbidities, acute condition, and length of mechanical ventilation. Oxygenation deficits were categorized using the oxygenation index (OI) or oxygen saturation index (OSI) as previously described in the PALICC-2 definitions of PARDS.^1^ Severity of illness was assessed using the pediatric logistic organ dysfunction score as previously described.^18^

#### DNA Extraction and Analysis

Brushes were thawed on ice with 1:100 β-mercaptoethanol to reduce mucus disulfide bonds. The resulting suspension was then passed through a QIAShredder (Qiagen) and treated with DNAse. DNA and RNA were then extracted using an AllPrep DNA/RNA Mini-Kit (Qiagen). DNA was quantified using the Qubit Flex Fluorometer (ThermoFisher Scientific). The DNA was then sent to the CCHMC DNA core and run on an Illumina Infinium MethylationEPIC v2.0 microarray platform.

#### Bioinformatic and Statistical Analyses

##### Quality Control and Normalization

Raw iDAT files were imported and processed using the minfi^19^ and sesame^20^ packages in R.^21^ Data normalization was performed using the Subset-quantile Within Array Normalization (SWAN) method, which minimizes technical variability between probe types while preserving biological differences. DNAm levels were quantified as beta (β) values, calculated as the proportion of methylated signal (M) to the total signal (M + U + c), where constant c = 100 (*β* = *M*⁄(*M* + *U* + *c*)). Probes with cross-hybridization, high detection p-values (> 0.01, which likely represent signal not much above baseline), β > 0.8 or < 0.2 across all samples (likely representing technical artifact), and those near single-nucleotide polymorphisms were excluded from the downstream analyses.

##### Principal Component Analysis (PCA) and Hierarchical Clustering

Principal component analysis (PCA) was performed in R using the top 5% most variable CpG sites to assess global methylation patterns. Heatmaps based on beta values were generated using the pheatmap package, with rows representing CpG sites and columns representing samples. Hierarchical clustering was conducted using the stats package with Ward’s method and Euclidean distance. Sample annotations were incorporated along the x-axis to aid in the interpretation of clustering patterns.

##### Analysis of promoter-proximal and distal patterns of methylation

To assess promoter-proximal and distal DNA methylation patterns, a metaplot was generated by calculating average methylation levels within ±4 kilobases of annotated transcription start sites (TSS). Genomic intervals surrounding TSSs were defined and binned using bedtools, and overlapping CpG probes were identified accordingly. Mean beta values were then calculated for each bin using R, with data processing performed via the dplyr package and visualization using ggplot2. Beta values were aggregated separately for the two sample groups identified through principal component analysis and hierarchical clustering.

##### Analysis of differentially methylated CpG sites in the promoter and body of annotated genes

Beta values were transformed to M-values for improved statistical properties during linear modeling. Differentially methylated CpG sites (DMCs) between the two groups were identified using the limma^22^ package. A linear model with empirical Bayes moderation was applied, with group assignment (1 vs 2) as the main covariate. CpGs with an adjusted p-value < 0.05 and absolute delta beta (Δβ) ≥ 0.1 were considered significantly differentially methylated. CpG sites exhibiting a ≥10% change (Δβ-value) in DNAm relative to controls were classified as hypermethylated (≥10% increase) or hypomethylated (≥10% decrease). EPIC probes located 1.5 kb upstream to 200 bp downstream of the transcription start site (TSS) were identified as promoters. Probes extending from the end of the promoter region to the 3′ end of the gene were classified as gene bodies. Probes outside promoters or gene body regions were designated as intergenic.

##### Pathway Enrichment Analysis:

Pathway analysis was performed separately for hyper- and hypomethylated genes mapped from differentially methylated promoter and gene body CpGs. Gene sets were annotated using the MSigDB v7.5.1 database, including hallmark, canonical pathways (C2), and gene ontology (C5) collections. Overrepresentation analysis (ORA) was conducted using the clusterProfiler package in R, which applies hypergeometric testing to assess the enrichment of gene sets. Background genes included all genes mapped from high-confidence CpG probes after filtering. Pathways with an adjusted p-value < 0.05 (Benjamini–Hochberg correction) were considered significantly enriched. Results were visualized using dot plots and bar plots generated with the ggplot2 package.

##### Biostatistical Analyses:

All biostatistical analyses were completed using R (v 4.5.0, The R Project for Statistical Computing). All statistical analyses were completed using base R functions. Continuous variables were non-normally distributed and were thus analyzed using the Mann-Whitney U test. Categorical variables were analyzed using Fisher’s exact test. A p-value cutoff of less than 0.05 was considered statistically significant.

## Results:

### Characterization of global DNA methylation patterns in PARDS and control subjects

**Figure 1.**
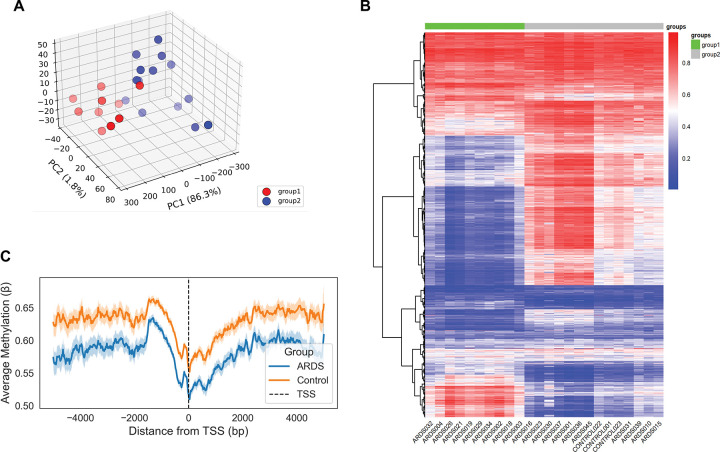
Clustering of ARDS and control samples based on DNA methylation profiles **(A)** Principal component analysis of methylation profiles. PCA was performed on the top 5% most variable CpG sites across all samples using beta values derived from Illumina EPIC V2 array data. The first two principal components were used to visualize sample clustering based on global methylation variation. **(B)** Heatmap with hierarchical clustering of highly variable CpG sites. A heatmap was generated using beta values from the same top 5% most variable CpG sites. Rows represent CpG sites, and columns represent individual samples. Hierarchical clustering was performed on rows and/or columns using Ward’s method and Euclidean distance. **(C)** DNA methylation landscape around transcription start sites (TSS). Metaplot showing average DNA methylation levels within ±4 kilobases of annotated TSS regions. CpG sites were binned relative to the TSS, and mean beta values were computed across all transcripts for Group 1 and Group 2 separately. The vertical dashed line marks the TSS (0 bp).

There were 170 patients screened, and a total of 24 specimens were analyzed, including 21 PARDS and 3 control subjects **(Supplemental Figure 1)**. There were 10 residual specimens from our previous work and 14 new samples (Supplemental Table 1). Principal component analysis and hierarchical clustering were performed to explore the methylation patterns of the top 5% most variable CpG sites (Figure 1A–C) across PARDS and control subjects. PCA revealed two distinct clusters of subjects, designated as Group 1 and Group 2. Group 1 (n = 10) contained only PARDS subjects, while Group 2 (n = 14) included all three controls and 11 PARDS subjects. Hierarchical clustering confirmed the findings, revealing two distinct groups. Group 1 again consisted only of PARDS patients, while Group 2 included some PARDS subjects and all three controls. We then examined promoter-proximal and gene body methylation levels using a metaplot of average DNA methylation levels within ±4 kilobases of annotated transcription start sites (TSS) (Figure 1C). This showed that Group 2 subjects had globally higher levels of methylation compared to Group 1 subjects. Both groups exhibit a dip in methylation around the TSS, as expected. We then examined the group assignments of the residual samples to determine whether they were consistent with those from our previous work. We found that 5 subjects maintained their group assignment, meaning they were in the lower risk groups in both studies **(Supplemental Table 1).** The other 5 subjects switched group assignments between studies, meaning they were high risk in one study and low risk in the other, or vice versa.

### Clinical characteristics of the subgroups

**Figure 2.**
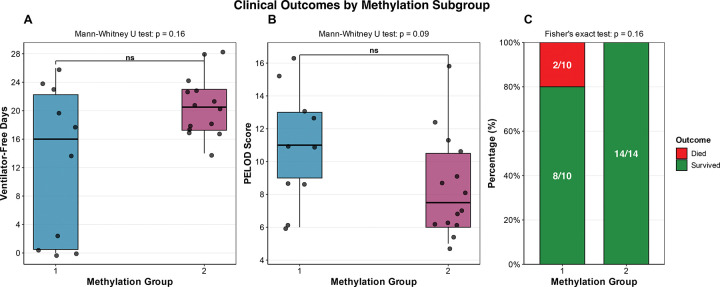
Clinical outcomes stratified by methylation group **(A)** Box plot showing the median number of ventilator-free days between the two groups. Group two had more ventilator-free days; however, this was not statistically significant. **(B)** Box plot showing the median PELOD scores between the two groups. Group 1 had a higher median PELOD score but this was not statistically significant. **(C)** The proportion of each group that survived or died. Both patients who died were in group 1; however, this result did not reach statistical significance.

The clinical characteristics of the subjects in each group were evaluated (Table 1). There was no difference in age, sex, or race between Group 1 and Group 2. Group 1 had a median of 16 VFDs, compared to 20.5 for Group 2; however, this difference was not statistically significant (p = 0.158). There was a large variance in VFDs in group 1 (Figure 2A). The PELOD scores were higher in group 1 (Figure 2B), although this did not reach statistical significance. There was no difference in primary comorbidity, acute condition, COVID status, direct lung injury, or infectious agent between the two groups. We then examined the outcomes and found that the two subjects who died were both in group 1 (Figure 2C). This finding did not reach statistical significance.

### Pathway analysis of differentially methylated CpG sites between groups

**Figure 3.**
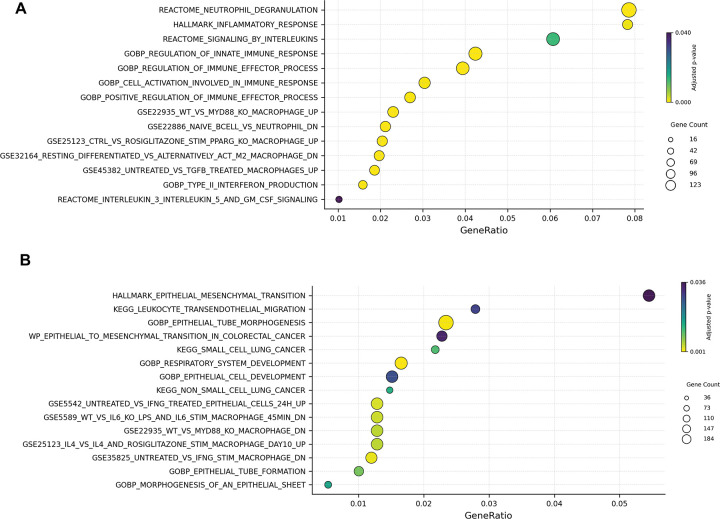
Pathway enrichment analysis of differentially methylated CpG sites in Group 2 relative to Group 1 **(A)** Enriched pathways among hypermethylated promoter CpG sites. Over-representation analysis (ORA) was performed on genes with significantly hypermethylated promoter CpG sites (adjusted p < 0.05, Δβ ≥ 0.1). Enriched pathways included immune regulatory processes such as *REACTOME_neutrophil_degranulation*, *GOBP_leukocyte_cell–cell_adhesion*, and *GOBP_regulation_of_T_cell_activation*. **(B)** Enriched pathways among hypomethylated promoter CpG sites. Genes with hypomethylated promoter regions were enriched in pathways related to epithelial structure and ion homeostasis, including *KEGG_focal_adhesion*, *REACTOME_extracellular_matrix_organization*, and *GOBP_sodium_ion_transport*.

We then evaluated patterns of differentially hyper- and hypo-methylated CpG sites in the promoters of annotated genes (FDR p-value <0.05 and Δβ ≥ 0.1) between the two groups. We found that Group 2 exhibited hypermethylation of many promoters flanking genes involved in innate immune pathways, including those related to neutrophil degranulation and interleukin signaling (Figure 3A). Conversely, group 2 exhibited hypomethylation of genes involved in the mesenchymal-to-epithelial transition and epithelial regeneration (Figure 3B). Taken together, this suggests that Group 2 shows a downregulation of the innate immune response, accompanied by an upregulation of mechanisms involved in repair and regeneration.

**Figure 4.**
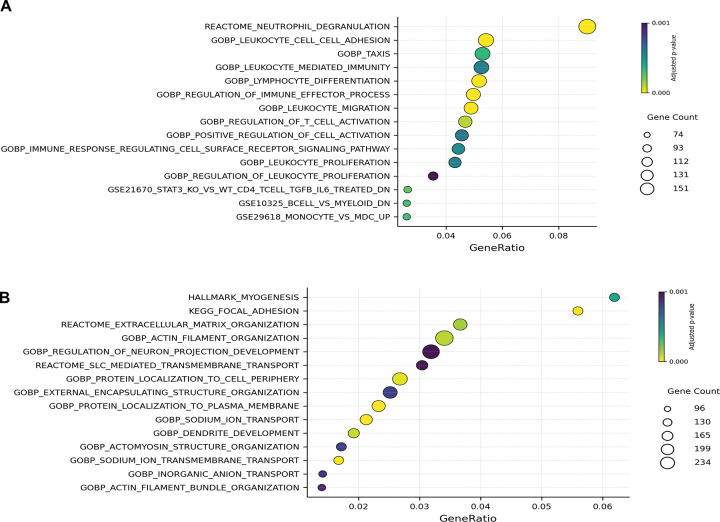
Pathway enrichment analysis of differentially methylated CpG sites in Group 2 relative to Group 1 **(A)** Functional enrichment of hypermethylated gene body CpG sites. Hypermethylated gene bodies in ARDS samples were associated with immune-related pathways, including *leukocyte migration* and *immune effector processes*. **(B)** Functional enrichment of hypomethylated gene body CpG sites. Gene bodies showing hypomethylation were enriched for pathways involved in extracellular matrix organization, cell adhesion, and cytoskeletal regulation. These epigenetic changes may reflect transcriptional programs supporting tissue remodeling and epithelial adaptation in the injured lung.

We then examined patterns of differentially hyper- and hypomethylated CpG sites in the bodies of annotated genes (FDR p-value <0.05 and Δβ ≥ 0.1) between the two groups. Group 2 exhibited increased hypermethylation of genes involved in the innate immune response, including those related to neutrophil degranulation and leukocyte adhesion, migration, and proliferation (Figure 4A). Group 2 had hypomethylation of genes involved in myogenesis and cell structural modifications (Figure 4B). These results are consistent with the methylation patterns observed in promoters, suggesting that group 2 downregulates many pathways involved in the innate immune response and upregulates pathways essential for repair and regeneration.

## Discussion:

In this study, our findings were mixed. We uncovered two groups of PARDS subjects that differed in the pattern of methylation of promoters and gene bodies involved in inflammation and the immune response. However, half of our 10 specimens analyzed in both our original work and this study switched between risk groups, which is concerning. There are several potential explanations for this. First, this study contains more subjects overall and adds 14 new subjects. Adding new subjects could easily change how the previous subjects clustered. For example, suppose new patients exhibit even higher levels of hypomethylation around inflammatory genes. In that case, subjects previously considered high risk in the previous cohort may now be classified as low risk. Another significant difficulty in directly comparing the studies is the use of different methodologies to analyze the methylome. The specific microarray technology (Infinium MethylationEPIC v2.0) used in this study was not available at the time of our original work. A microarray is a less labor-intensive methodology with greater translational potential.

In our current study, Group 2, compared to Group 1, exhibited hypermethylation of both promoters and bodies of genes involved in inflammation and immunity, and hypomethylation of genes associated with the epithelial-mesenchymal transition. Group 2 also showed a trend toward better clinical outcomes compared to Group 1, although this difference was not statistically significant. This finding is broadly consistent with our previous work, which described two groups of PARDS patients that differed in the pattern of methylation in promoters flanking genes regulating the innate immune response.^16^ In that cohort, our findings were very similar, including hypomethylation of promoters flanking genes involved in the innate immune response in the subgroup of patients that trended toward worse clinical outcomes.

The findings in both studies suggest that the group at higher risk of adverse clinical outcomes has epigenetic changes suggestive of upregulation of genes governing the inflammatory response. Categorizing PARDS by epigenetic signatures has promise both as a strategy for prognostic enrichment and for identifying modifiable targets for new therapies.

There have been many studies examining the methylome in pediatric asthma,^12,13,23,24^ adult asthma,^25–28^ and adults with COPD.^14,29,30^ However, fewer studies have examined the contribution of the methylome in adult ARDS subjects, and no studies have been conducted in the pediatric ARDS population. Several previous studies have investigated the role of the methylome in COVID-19. One group demonstrated that in adult COVID-19 subjects with ARDS, mutations in DNA methyltransferase 3 alpha (DNMT3A) were associated with worse outcomes, and this effect was likely mediated through increased transcription of interleukin-32 (IL-32). ^31^ Another group showed that survivors of COVID-19 developed hypermethylation of pathways linked to the immune response, such as tumor necrosis factor alpha (TNF-α), IL-2, and IL-6, while subjects who died did not. Finally, a study using epigenome-wide association analysis (EWAS) of peripheral blood samples from COVID-19 patients built a model that predicted disease status and severity.^32^ They found that the best model included CpG sites related to immune pathways, specifically the interferon response. Taken together, these data are consistent with our findings, suggesting that the methylome may play a crucial role in regulating the immune response to ARDS.

Our finding that group 2 subjects had hypomethylation of pathways involved in the repair and regeneration pathway is intriguing. The process of lung repair and regeneration shares many similarities with the process of lung development,^33^ and the importance of epigenetic regulation of both processes has been previously demonstrated. The importance of epigenetic regulation is evident during the early stages of development, as the regulation of chromatin accessibility has been shown to be crucial during the differentiation of endoderm into lung progenitors.^34^ The regulation of chromatin accessibility by the histone lysine-methyl transferase proteins PRDM3 and 16 was also shown to be critical for alveolar type 1 (AT1) and alveolar type II (AT2) cells, the primary epithelial cells of the alveolus.^35^ Regulation of repair has also been shown to rely heavily on chromatin accessibility as it has been shown that the normal AT2 to AT1 cell transition that occurs in response to injury is governed by a chromatin state regulated by Nkx2–1.^36^ Taken together, these findings suggest that epigenetic regulation may represent one regulatory mechanisms that explains the differential host response to seemingly similar insults.

Our work is the first to evaluate the methylome and its impact in PARDS. Despite our mixed results, we do provide evidence that epigenetic regulation may be an important risk factor for PARDS outcomes and deserves further study. Future studies should focus on enrolling significantly more subjects and running parallel enzymatic methyl-seq and the Infinium microarray to determine how the two methods correlate and validate this approach.

There are several weaknesses to our study. First, our sample size is small, which limits our ability to detect significant differences in the clinical outcomes of the subjects. Second, we did not identify epigenetic changes at the single-cell level and thus cannot determine whether the differential methylation patterns we observe are primarily present in immune cells, epithelial cells, or both. There are currently technologies under development that will enable the analysis of the methylome at the single-cell level; however, they are not yet available. Thirdly, the single-center nature of our study could limit the generalizability of these findings pending confirmation from a multi-center cohort. Finally, the most significant limitation is that half of the subjects in both cohorts switched risk assignment.

## Conclusion:

We identified two subgroups of PARDS subjects, defined by differential methylation of the promoters and bodies of genes involved in the immune response, repair, and regeneration. Further study of the epigenetic regulation of the immune response to PARDS is warranted as a potential therapeutic target.

## Supplementary Material

Supplementary Files

This is a list of supplementary files associated with this preprint. Click to download.

• SupplementalTable1.docx

• SupplementalFigure1.docx

## Figures and Tables

**Table 1:** Demographic and Clinical Characteristics by Methylation Subgroup Demographic and clinical variables stratified by methylation group assignment. For continuous variables, values are expressed as median and the 25%-75% interquartile range. For categorical variables, values are expressed as proportions. Mann-Whitney U test and Fischer’s exact test were used to analyze continuous and categorical data, respectively.

Characteristic	Methylation Group 1 (N=10)	Methylation Group 2 (N=14)	P-value

**Age, years**	**6.4 (2.5, 8.6)**	**8.1 (4.5, 9.9)**	**0.464**
**Sex**			
*Female*	*4 (40.0)*	*6 (42.9)*	*1.000*
*Male*	*6 (60.0)*	*8 (57.1)*	
**Race**			
*AA*	*1 (10.0)*	*2 (14.3)*	*1.000*
*Non-White*	*1 (10.0)*	*2 (14.3)*	
*Unknown*	*0 (0.0)*	*1 (7.1)*	
*White*	*8 (80.0)*	*9 (64.3)*	
**PELOD Score**	**11.0 (9.0, 13.0)**	**7.5 (6.0, 10.5)**	**0.086**
**Ventilator-Free Days**	**16.0 (0.5, 22.2)**	**20.5 (17.2, 23.0)**	**0.158**
**PARDS Severity**			
*None*	*0 (0.0)*	*2 (14.3)*	*0.077*
*Mild*	*1 (10.0)*	*6 (42.9)*	
*Moderate*	*7 (70.0)*	*3 (21.4)*	
*Severe*	*2 (20.0)*	*3 (21.4)*	
**Principal Comorbidity**			
*Developmental Delay*	*0 (0.0)*	*1 (7.1)*	*0.610*
*Endocrine*	*1 (10.0)*	*0 (0.0)*	
*Genetic Syndrome*	*1 (10.0)*	*4 (28.6)*	
*GI*	*0 (0.0)*	*2 (14.3)*	
*Hematologic*	*0 (0.0)*	*1 (7.1)*	
*Immunocompromised*	*3 (30.0)*	*1 (7.1)*	
*Neurologic*	*1 (10.0)*	*1 (7.1)*	
*None*	*2 (20.0)*	*2 (14.3)*	
*Oncologic*	*0 (0.0)*	*1 (7.1)*	
*Pulmonary*	*2 (20.0)*	*1 (7.1)*	
**Acute Condition**			
*Encephalitis*	*1 (10.0)*	*0 (0.0)*	*0.090*
*Pneumonia*	*3 (30.0)*	*8 (57.1)*	
*Post Surgical*	*0 (0.0)*	*3 (21.4)*	
*Sepsis*	*4 (40.0)*	*1 (7.1)*	
*Shock*	*1 (10.0)*	*0 (0.0)*	
*Trauma*	*1 (10.0)*	*2 (14.3)*	
**COVID**			
*No*	*9 (90.0)*	*13 (92.9)*	*1.000*
*Yes*	*1 (10.0)*	*1 (7.1)*	
**Direct Lung Injury**			
*No*	*4 (40.0)*	*6 (42.9)*	*1.000*
*Yes*	*6 (60.0)*	*8 (57.1)*	
**Infectious Agent**			
*Bacteria*	*2 (20.0)*	*3 (21.4)*	*0.743*
*None*	*3 (30.0)*	*7 (50.0)*	
*Virus*	*3 (30.0)*	*3 (21.4)*	
*Virus and Bacteria*	*2 (20.0)*	*1 (7.1)*	
**Outcome**			
*Died*	*2 (20.0)*	*0 (0.0)*	*0.163*
*Survived*	*8 (80.0)*	*14 (100.0)*	

Data presented as median (25th, 75th percentile) for continuous variables and n (%) for categorical variables.

Statistical tests: Mann-Whitney U test for continuous variables; Fisher's exact test for categorical variables.

VFD = Ventilator-Free Days at 28 days. P-values <0.05 considered statistically significant.

## Data Availability

The raw IDAT files, metadata, microarray experimental design with sample data relationships, and probe sequences are publicly available on the National Institutes of Health (NIH) Gene Expression Omnibus (GSE 303363). The metadata used to calculate differences in clinical outcomes contain protected health information (PHI) and are not able to be posted publicly.
